# Sociocultural and epidemiological aspects of HIV/AIDS in Mozambique

**DOI:** 10.1186/1472-698X-10-15

**Published:** 2010-06-08

**Authors:** Carolyn M Audet, Janeen Burlison, Troy D Moon, Mohsin Sidat, Alfredo E Vergara, Sten H Vermund

**Affiliations:** 1 Vanderbilt Institute for Global Health, Vanderbilt University School of Medicine, Nashville, TN, 37203 USA; 2 Department of Preventive Medicine, Vanderbilt University School of Medicine, Nashville, TN, 37232 USA; 3 Department of Pediatrics, Vanderbilt University School of Medicine, Nashville, TN, 37232 USA; 4 Friends in Global Health, Maputo and Quelimane, Mozambique; 5 Department of Community Health, Faculty of Medicine, Universidade Eduardo Mondlane, Maputo, Mozambique

## Abstract

**Background:**

A legacy of colonial rule coupled with a devastating 16-year civil war through 1992 left Mozambique economically impoverished just as the human immunodeficiency virus (HIV) epidemic swept over southern Africa in the late 1980s. The crumbling Mozambican health care system was wholly inadequate to support the need for new chronic disease services for people with the acquired immunodeficiency syndrome (AIDS).

**Methods:**

To review the unique challenges faced by Mozambique as they have attempted to stem the HIV epidemic, we undertook a systematic literature review through multiple search engines (PubMed, Google Scholar™, SSRN, AnthropologyPlus, AnthroSource) using Mozambique as a required keyword. We searched for any articles that included the required keyword as well as the terms 'HIV' and/or 'AIDS', 'prevalence', 'behaviors', 'knowledge', 'attitudes', 'perceptions', 'prevention', 'gender', drugs, alcohol, and/or 'health care infrastructure'.

**Results:**

UNAIDS 2008 prevalence estimates ranked Mozambique as the 8^th ^most HIV-afflicted nation globally. In 2007, measured HIV prevalence in 36 antenatal clinic sites ranged from 3% to 35%; the national estimate of was 16%. Evidence suggests that the Mozambican HIV epidemic is characterized by a preponderance of heterosexual infections, among the world's most severe health worker shortages, relatively poor knowledge of HIV/AIDS in the general population, and lagging access to HIV preventive and therapeutic services compared to counterpart nations in southern Africa. Poor education systems, high levels of poverty and gender inequality further exacerbate HIV incidence.

**Conclusions:**

Recommendations to reduce HIV incidence and AIDS mortality rates in Mozambique include: health system strengthening, rural outreach to increase testing and linkage to care, education about risk reduction and drug adherence, and partnerships with traditional healers and midwives to effect a lessening of stigma.

## Background

**Figure 1:**
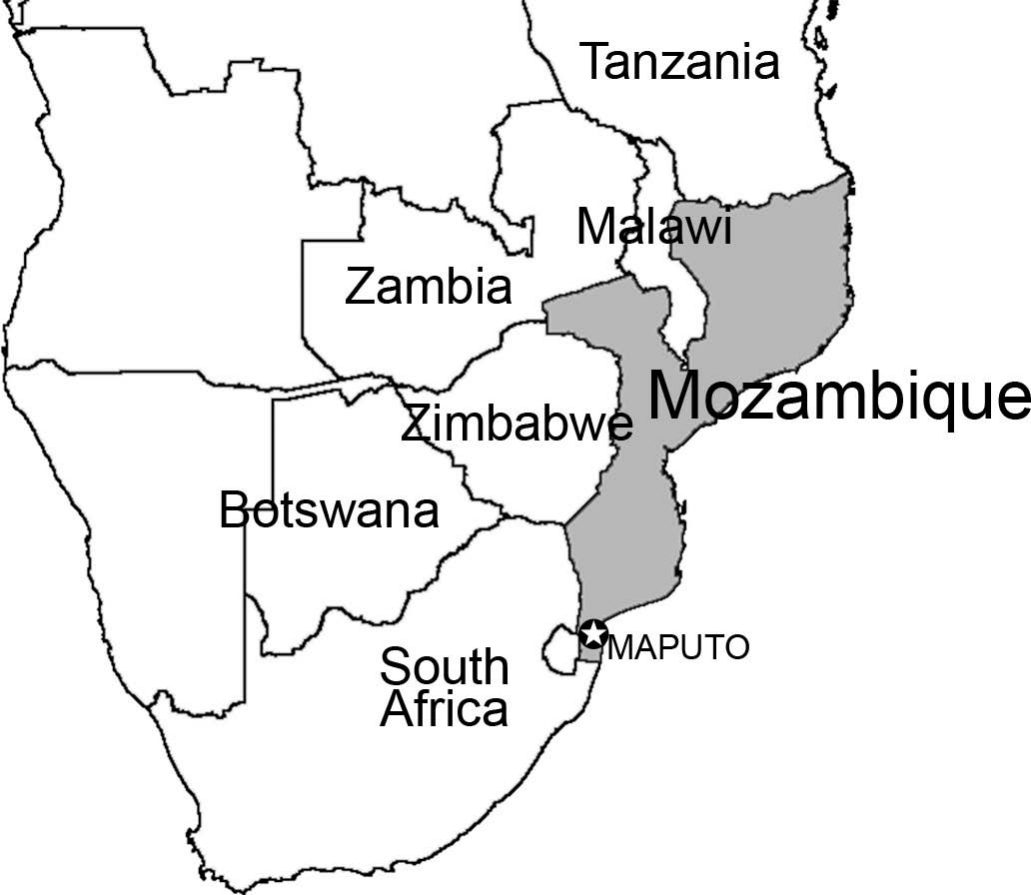
**Map of Mozambique and surrounding countries**.

Mozambique, a southeast African nation of ≈21 million people, suffers one of the world's highest burdens of human immunodeficiency virus (HIV) and acquired immunodeficiency syndrome (AIDS) (Figure 1). In 2007, HIV prevalence in the 36 antenatal clinic (ANC) sentinel surveillance sites ranged from 3% to 35% with a national estimate of 16% (plausibility bounds from 14-17%) in women ages 15-49 years [1]. Provincial HIV prevalence estimates ranged from 8% to 27% and were highest in the central and southern provinces [1]. The impact of the 10-year armed struggle for independence from the Portuguese (1964-1974) and the 15-year externally financed insurgency (1977-1992) resulted in the devastation of industrial and governmental infrastructures, including health clinics and schools [2]. The end of war brought increased social and economic stability, but coincided with rising HIV/AIDS rates as Mozambique's national isolation ended.

The first case of HIV was reported in Mozambique in 1986 [3]. By 2001, one million people were estimated to be living with HIV, including 53,000 children under the age of 18 and 570,000 women [1]. In response, the MISAU developed a strategic framework focusing efforts on reduction of vertical transmission, prevention of HIV transmission in health facilities for nosocomial and occupational exposures, and improved access to life-saving treatment through expansion of its antiretroviral therapy (ART) program. Prior to 2004, ART was available only through pilot programs, with the majority of the population given cotrimoxazole prophylaxis and medicine to manage opportunistic infections. ART became available nationwide in 2004 with the support of international funding agencies. Despite these efforts, < 25% of those with advanced HIV disease were receiving treatment in 2007. An estimated 1.5 million people were living with HIV in Mozambique in 2007, including 810,000 women and 100,000 children [1]. Annual deaths from AIDS-related causes are estimated to have increased from approximately 45,000 people in 2000 to 97,000 in 2005 [4].

HIV prevalence varies markedly by region, sex, and age [1]. An estimated 58% of adults living with HIV in 2007 were women, with fully three-quarters of the infected 15-19 year olds being female (8.5% prevalence in females and 2.9% in males) [1]. The higher prevalence in females may reflect a greater biological susceptibility to the disease, but is assuredly exacerbated by a multitude of cultural and social factors; women are offered HIV testing more regularly than men due to pregnancy and programs aimed to prevent vertical transmission of HIV [1,4,5]. This paper is designed to provide readers with an overview of the unique challenges to HIV prevention and treatment in Mozambique through a review of the sociological, anthropological, and epidemiological literature published relating to the HIV/AIDS epidemic.

## Methods

We undertook a systematic literature review through multiple search engines (PubMed, Google Scholar™, SSRN, AnthropologyPlus, AnthroSource) using Mozambique as a required keyword. We searched for any articles that included the required keyword as well as the terms 'HIV' and/or 'AIDS', 'prevalence', 'behaviors', 'knowledge', 'attitudes', 'perceptions', 'prevention', 'gender', drugs, alcohol, and/or 'health care infrastructure'. Further, we reviewed the citations of each article to identify other appropriate reports. Unpublished reports were requested from the Centers for Disease Control and Prevention (CDC) in Mozambique, the Joint United Nations Programme on HIV/AIDS (UNAIDS), the United Nations Children's Fund (UNICEF), the World Health Organization (WHO), and the Mozambique Ministry of Health (MISAU). We reviewed all articles published since 1990 that we felt were relevant (n = 100 articles from total 130 with Mozambique and HIV or AIDS as key words) based on their epidemiological and/or behavioral data. We report here those key published data and observations that we felt relevant to impact of HIV in Mozambique. Our systematic literature review of the Mozambican HIV/AIDS epidemic highlights challenges to health care professionals, policy makers, and those providing social and education programming to the socio-cultural, anthropological and infrastructural challenges impeding HIV prevention and treatment.

## Results

Unprotected heterosexual sex and vertical transmission are known risk factors for HIV infection in Mozambique [1]. However, neither male-to-male sexual contact nor use of unsafe needles has been studied adequately. Despite having infection rates that are among the highest in the world, individual risk perceptions among Mozambicans underestimate personal risk markedly [6,7]. In 2004, young women in Maputo demonstrated substantial difficulty in assessing their own risk even when they had correct knowledge about HIV transmission [8].

High risk activities, among both men and women, may be encouraged by local traditions and customs [9]. These include cultural rituals and norms such as 'widow cleansing', in which a brother-in-law or other male relative has sex with the wife of the deceased family member, early sexual initiation, intergenerational and transactional sex, and intravaginal practices such as vaginal instillation of desiccant herbs for 'dry sex' [5,10-13].

An underdeveloped primary care system and chronic health manpower shortages could potentially contribute to HIV transmission risk. For example, if sterile needles and syringes are not used for vaccination and health care HIV can be transmitted. Similarly, blood transfusions may transmit HIV in the absence of an excellent national blood banking system. Untreated co-infections such as tuberculosis, malaria, sexually transmitted infections (STIs), and geohelminthes can increase the likelihood of HIV transmission though dysregulation of host immunity and subsequent spikes in viral load (in HIV-infected) and immune activation (in both HIV-infected and uninfected); this can increase susceptibility of HIV-uninfected and disease progression and infectiousness of HIV-infected persons [14,15].

### Heterosexual Risk Behavior

**Figure 2:**
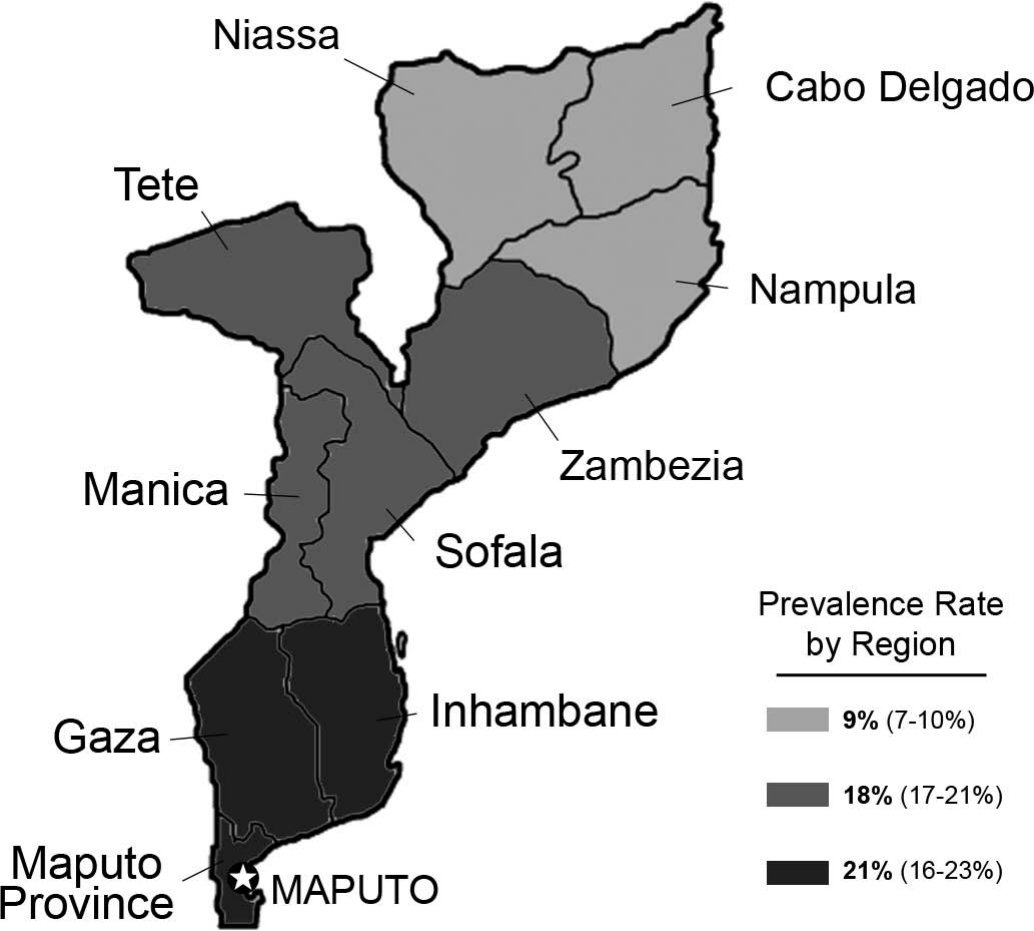
**Map of Mozambique highlighting regional prevalence rates**.

A high prevalence of STIs, early age at first sexual experience, and the culturally accepted practice among some sub-groups to have multiple, concurrent partners are thought to substantially increase HIV transmission risk [16]. In 2007, the MISAU found median syphilis rates (by RPR) to be 3% in clinics in the southern provinces (range 1-18%), 7% (1-16%) in the central provinces, and 12% (1-26%) in the northern provinces; syphilis rates correlated poorly with regional HIV infection rates (Figure 2) [1]. HIV infection rates are higher in the southern and central parts of the country, with lower rates in the north. A 2007-2008 the MISAU study conducted in the city of Xai-Xai in Gaza Province and in Maputo documented a high prevalence of STIs in HIV-infected people on their first care visit, including syphilis (15.2% were RPR and TPHA-positive), trichomoniasis (10.2% in men and 48.5% in women by PCR), bacterial vaginosis (38.6% by Nugent's criteria), and vaginal candidiasis (10% on Gram stain) [17]. Notably, 86.8% of men and 94.8% of women were herpes simplex virus-2 (HSV-2) positive on serology [17].

Early sexual debut is common in Mozambique, with one survey reporting that 74% of Mozambican women had had their first sexual experiences before age 18 [18]. A 2002 national survey of reproductive health among 15-24 year olds reported that 24% of females and 30% of males had intercourse before age 15 [5]. In the 2003 Demographic and Health Survey (DHS), the median age of first intercourse was 16.1 years for women and 17.8 for men [19]. Sexually active young women in neighboring Zambia reported having sex with older partners who were potentially at higher risk for HIV infection, and it is likely that young Mozambican women are engaging in similar behavior [20]. Given that younger women may have a higher biological risk for contracting HIV and STIs due to presence of cervical ectopy (non-keratinized exocervical tissue), the benefits of delayed sexual initiation and condom use are especially important for adolescents [21].

### Socio-economic Diversity

Distinctions in socioeconomic status, access to education, language, and gender relationships divide the northern, central and southern parts of the country. Despite its status as the official language of Mozambique, in 2003 Portuguese was spoken fluently by only 40% of the population [19]. Of the 42 languages spoken in Mozambique, 41 derive from the Bantu language family [22]. Reflecting the ethnic incoherence of colonial divisions that now constitute the borders of modern African nations, neighboring countries with strong linguistic links (examples are given) include South Africa (Zulu, Tsonga), Zambia (Nyanja, Nsenga), Malawi (Chichewa, Yao), Zimbabwe (Tsonga, Ndau), Swaziland (Swati, Tsonga), and Tanzania (Yao, Makonde). Mozambicans frequently migrate to South Africa and other nations of high HIV endemicity for both short- and long-term job opportunities [22,23]. This migration presents a challenge for the government, partner non-governmental organizations (NGOs), and faith-based organizations (FBOs). For example, Tonga men commonly migrate to South Africa to work in the mines for several years at a time, a well documented context for increased risk for themselves and their families [23]. Health care services and education for miners and their families have been limited, however [23]. As Mozambicans living in southern and central parts of the country are those mostly likely to migrate, this may contribute to high HIV infection rates.

Securing money to stay in school and/or buy goods or food has been identified as a reason for adolescent girls in sub-Saharan Africa to participate in sexual relationships [24-26]. Girls in the capital city of Maputo may engage in sexual relationships with older men in efforts to increase their social status, which includes monetary support for educational opportunities and for material goods [12]. In Mozambique, as in much of the rest of Africa, women are far less financially secure than men, earning 18% of the typical salary of a man [19]. Financial dependence on men is exacerbated by low rates of secondary school completion among females, low incomes for women in rural areas, and limited employment choices for uneducated individuals other than subsistence agriculture and trading [19]. A number of studies have shown that educating women both reduces risky sexual behaviors and increases their economic and social options [3,27].

### Vertical Transmission

Vertical transmission is mitigated by proper access to antenatal care that includes HIV testing and access to ART [28]. Without vertical transmission prevention programs, 25-48% of women will pass the virus on to their children during pregnancy, childbirth, or breastfeeding [29,30]. Nationwide vertical transmission prevention program coverage is inadequate, with easy access to nevirapine and zidovudine limited primarily to urban and district hospitals. The Drugs, Resources Enhancement against AIDS and Malnutrition (DREAM) project supported vertical transmission prevention implementation resulting in only a 4% transmission rate [31].

Breastfeeding is a major cause of vertical transmission in Mozambique, where replacement feeding is unaffordable for the vast majority of women. Even when provided free of charge, sociocultural norms support continued breastfeeding; mixed feeding will result in more infections than exclusive breastfeeding alone [32]. Women rarely practice early weaning that would carry the stigma of being identified as sick or for fear of the nutritional status of the infant [33]. The latter concern has been validated in the Zambian Exclusive Breastfeeding Study in which infants were less likely to be HIV-infected on rapid weaning at age 4 months, but were more likely than breastfed babies to die from other causes [32]. In Mozambique, Malawi, and Tanzania, women who breastfed but were kept on ART had a reduced chance of passing the virus on to their children than those given formula; it was noted that mixed-feeding was common in the cohort given formula [33]. In early 2010, Mozambican health authorities are currently considering policy changes based on multinational studies supporting ART for breastfeeding HIV-infected mothers to reduce breast milk-acquired infection [34,35].

### Prevention Campaigns

Interventions aimed at modifying personal behaviors include campaigns to reduce sex among teens (abstinence), to reduce the number of sexual partners, and to encourage monogamy within marriage (be faithful), and promote correct and consistent condom use (the ABC campaign) [3,9,30,36,37]. Obstacles to condom use are many. A prevalent belief supported by the popular media is that condoms are not necessary in relationships built on mutual respect, love and trust [36]. Due to these messages, condoms may be more often used when having a sex with non-regular partners [9,30,36,37]. Health seeking behavior is also reportedly an issue, as many Mozambicans believe certain illnesses are caused by witchcraft or curses [38]. These beliefs prompt persons who are ill to seek care from traditional healers instead of seeking testing and treatment from a local health facility.

Interventions aimed at changing culturally accepted traditions often present the greatest challenge because, unlike many structural interventions, they are not easily modified by increased funding. Stigma, discrimination, and traditional gender roles present challenges when preventing and treating HIV. HIV-infected Mozambicans are typically afraid to be open about their HIV status for fear of community stigmatization and family abandonment [39]. Women have fewer opportunities to improve their financial position as they are less well educated and do not have the option of taking mining, construction or other 'hard-labor' jobs [39,40]. Where women and men are valued for the number of children they produce, condom use is eschewed even if a partner fears contracting the virus [41].

Structural interventions, based on international guidelines, have been implemented to reduce risk of transmission by eliminating the re-use of needles for immunizations and medication administration and from testing blood products used in transfusions [42,43]. One structural intervention to decrease HIV transmission is the provision of universal opt-out testing in health facilities which allows for earlier diagnosis and positive prevention counseling [44]. Opt-out testing was adopted as protocol by the Mozambican Ministry of Health for pregnant women in 2006 [45]. Potential human rights issues and stigmatization of women that may result from the introduction of this strategy are detailed in the Discussion section.

Educational and health systems, particularly in rural areas, were devastated by the long periods of war; reconstruction has been slow since peace in 1992 due to inadequate funds in the face of devastation of both schools and hospitals [46]. In addition to the improvement of education and health facilities, the creation and enforcement of laws protecting women's rights are needed. One example is Mozambique's law to protect women from losing homes and property after the deaths of their husbands; despite enaction it has been enforced infrequently [47]. Property, including clothing, land, and homes will often be seized by the family of the deceased, ostensibly to ensure that it remains in the biological family of the deceased man.

### Adherence to ART

A recent qualitative study of barriers to ART in Beira, Mozambique found that patient information and attitudes towards treatment, family support, and clinical confidentiality were all important barriers to health seeking behavior among those infected with HIV [48]. Assuming service availability, there are still issues regarding clinician-patient exchange (including confidentiality, respect and education), as well as community interventions to encourage support for HIV positive individuals within their families and communities. Poor adherence presents viral resistance, treatment failure, and higher viral loads, leading to increased viral transmission [49]. Thus, adherence is essential to any treatment program.

Two long-term studies of patient adherence have been undertaken in urban communities in Mozambique. A study of 154 people treated by the privately-funded DREAM program suggested that 82.5% of people were > 90% adherent to their treatment, as estimated by the proportion that kept their ART-related appointments and maintaining lowered viral loads. Surveyed patients indicated that the visits and medicines, both free of charge nationwide, and nutritional supplements, available free in the DREAM program, but not nationwide in HIV care venues, were the most important factors in adherence. A second study, also at a DREAM clinic, suggested that 72.1% of patients had a >95% adherence. Only 5% were believed to be adherent < 85% of the time [33]. Although these numbers from a well-funded model program are encouraging, they are not representative of national ART services that include rural areas hampered by limited budgets, transportation, extreme poverty, public financing, limited health workforce, and poor population educational levels. Systematic reviews of ART programs throughout Mozambique are needed within their broader health care context.

### Drug and Alcohol Use

Little is known about drug and alcohol use in Mozambique. Most Mozambican communities are neither prosperous enough to sustain drug purchases nor located in any illicit drug distribution corridor. However, Maputo, Beira, and other smaller cities are potential ports where drugs could be imported to South Africa or Zimbabwe. Elsewhere in sub-Saharan Africa drug use of heroin, Mandrax, methamphetamine and cocaine has been rising [50,51]. The implications of increased HIV transmission through infected needles and increased risky behavior with the expansion of illicit drug use has been noted in South Africa, Kenya, Tanzania, Mauritius, and Nigeria [52,53]. At present, it is unknown whether drug use contributes to HIV infection in Mozambique's cities such as Maputo or Beira; we believe it is an important area for future research.

Alcohol use has been associated with HIV infection risk in both sub-Saharan Africa and other countries due to disinhibition and increased sexual risk-taking [54-59]. There is no published evidence of a relationship between alcohol use and HIV transmission in Mozambique. Homemade alcoholic beverages from cashew fruit, corn, and sugarcane are the cheapest type of alcohol available in rural areas and beer is widely available. We believe that it is highly plausible that there are links, and as with drug use, alcohol consumption is an important future HIV research priority.

### Men Who Have Sex with Men (MSM)

MSM, male homosexuality, and bisexuality are taboo topics and practices in southern Africa. Nonetheless, key informant interviews with men living in Mombasa and Nairobi, Kenya identified an underground community of MSM [60]. It is likely that MSM exist also in southern African cities, but because of the stigma associated with homosexuality, locating people willing to discuss their homosexual sexual behavior is challenging. Imprisonments in 2009 of MSM in Uganda and Malawi merely because the men were public as to their sexual orientation underscore MSM stigma, discrimination, and human rights issues[61]. While not illegal in Mozambique, the topic is taboo and homosexuality is often not accepted. We suggest that this topic represents yet another research vacuum for Mozambique.

### Cultural Practices

Some local cultural practices may place individuals at an increased risk for contracting HIV, but care must be taken to not overstress 'exotic' or 'unacceptable' behavior as seen by foreign researchers [62]. Rates of adult HIV infection are highest in the southern and central parts of the country (21% and 18%, respectively) compared to 9% in the Northern provinces [1]. It is plausible that some socio-cultural differences may be influencing HIV prevalence rates. These include marriage practices, sexual initiation that may occur during rites of passage, sexual practices linked to 'widow cleansing,' cutting the skin during traditional medical treatment, and desires by men to have 'dry sex' [10,12]. Although no systematic studies link these practices to HIV infection in Mozambique, the use of non-sterile knives and blades, unprotected sex, excoriation and other sexual trauma are obvious potential risk factors for transmission [63].

Anthropological studies undertaken in Mozambique to identify patterns in marriage structures (monogamy, polygamy), type of descent pattern (matrilineal, patrilineal), and age at marriage found significant variations in behavioral and social norms within the country's 16 major ethnic groups [64]. Outside of Mozambique, ethnic groups with patrilineal descent systems typically result in a younger age at first marriage for women and a larger age gap between a husband and a younger wife [65]. Unexpectedly, the opposite may be true in Mozambique. Matrilineal societies averaged earlier-than-expected ages for marriage (15-17 years of age) and patrilineal societies had a slightly higher average age at marriage (18-21 years) [66]. Education has likely become a mitigating factor, as matrilineal systems in the rural northern regions had high female illiteracy rates (85% - 88%). The southern provinces, where patrilineal descent is common, had lower rates of illiteracy (48% - 77%) and had greater access to radio, television, newspapers and health information [66]. No information for men was obtained in these studies such that we do not know about discrepancies in age at marriage between men and women.

Polygamy and systems of patrilineal descent are commonly practiced throughout the country [66]. The majority of people in the southern and central parts of the country are Christian. Conversely, the north is populated by a large percentage of Muslims [63]. In Muslim Africa, rates of HIV infection are typically lower than in other regions, probably due to the closed nature of polygamous sexual networks, lower incidence of premarital sex, and much higher rates of male circumcision [67].

'Widow cleansing' (a brother having perceived sexual access to his deceased brother's widow) is practiced in some Mozambican ethnic groups [68]. A widow is encouraged to have sexual relations with a brother of the deceased husband in an effort to "cleanse" her and "rid her of bad spirits." [68] She may also marry him in a polygamous or monogamous relationship. This ceremony marks the end of a woman's grieving period. A possible HIV-related cause of the death for the husband, HIV-infection in the brother, or HIV-infection in the widow may lead to HIV transmission.

Traditional medical beliefs and traditional healers may also play a role in spreading HIV infection and hindering HIV-related treatment. Traditional healers may exacerbate spread of the disease by prescribing sex with virgins to cure HIV, performing medical treatments with unsterilized blades, or having sex with their clients for alleged therapeutic benefit [69-81]. Perhaps more commonly, traditional healers may delay and discourage people from accessing HIV care and treatment in state-run medical clinics in order to continue treatment of infected individuals through traditional means [81]. Not all healers have negative relationships with the health care system, but there is currently no formal relationship between healers and the Ministry of Health to improve health care outcomes has yet been established.

Dry sex has been reported from many parts of southern Africa and can increase the chance of HIV transmission through abrasions of the vagina, vulva, cervix, or penis [5]. Women place drying agents like herbs, disinfectants, or soaps inside the vagina to ensure dryness during intercourse [5]. The origin of this practice may be associated with the belief that vaginal dryness during intercourse allows a man to maintain his erection for a protracted time [12].

## Discussion

### Prevention Challenges

There are many difficulties in integrating HIV prevention messages into the Mozambican context of stigma, low autonomy for women, influence of traditional healers, and a high cultural value of pregnancy. Even when HIV/AIDS knowledge is high, we think that culturally sanctioned methods for prevention, culturally adapted messages for HIV prevention and ART advocacy, and finding a creative way to integrate traditional healers into the health care system will be needed for long-term success in HIV programs. Lusophone Mozambique is distinctive compared to its six Anglophone neighbors. Both its Portuguese colonial and language heritage, and its troubled legacy of war and authoritarian government suggest that many prevention programs developed elsewhere will need adaptation for Mozambican conditions. Behavioral and structural interventions are not yet incorporated widely into national efforts. Among the needs are including more peer educators to dispense HIV testing and prevention information, increased access to ART, access to free condoms at truck stops, schools, clinics, and hotels, the creation and enforcement of laws protecting the legal rights of women, improved educational opportunities, and expanded male circumcision programs [82].

For the hundreds of thousands of serodiscordant couples in Mozambique and elsewhere, there is no effective method for them to protect themselves if they are not using condoms. Both men and women typically wish to have children due to a desire for a family, to meet social norms, and to indicate a successful marriage. These traditions surely challenge condom promotion within a relationship. Misconceptions about condoms may lead to paranoia or demagoguery, such as when a national church leader claimed AIDS to be a Western genocidal invention and that powder or lubrication in condoms caused HIV/AIDS [83]. So far, condoms use has be taken up by only a fraction of the population; other barrier methods like microbicides or ART used as pre-exposure prophylaxis (PrEP) that allow for pregnancy and a 'more authentic' feel during sex may have promise in the Mozambican setting. Of course, ART may reduce risk of transmission and is itself a potential prevention tool [84].

The adoption of opt-out testing only for pregnant women is complicated from a human rights perspective when community stigma is very high. Programs that overwhelmingly offer opt-out tested to pregnant women will naturally first find women to be positive, increasing serious social and marital pressures [85]. Ideally, a national strategy of opt-out testing in a high prevalence environment should be applied equally to both men and women whenever they visit a health provider regardless of chief complaint.

Despite an influx of funds from the President's Emergency Plan for AIDS Relief (PEPFAR) and the Global Fund to Fight AIDS, Tuberculosis, and Malaria (Global Fund), Mozambique remains woefully undercapacitated in its medical infrastructure, social acceptance of risk reduction strategies, and its health manpower [86]. Increased funding has improved HIV clinical services in much of urban Mozambique, but clinics in rural areas continue to have poor physical infrastructures and suffer from drug supply chain management issues. This creates a challenge for nurses, medical technicians, and doctors seeking to ensure universal access to counseling, testing, clinical care, stable ART access, ABC prevention services, and PMTCT. The dearth of health care providers is a deficit throughout sub-Saharan Africa. It is estimated that an additional 2.4 million health physicians, nurses, and midwives are necessary to meet universal health intervention coverage targets in the 57 most resource-constrained countries, notably Mozambique [87]. This discrepancy demands an urgent and realistic plan to increase production and retention of health care providers, particularly outside the capital city of Maputo where over half of small number of Mozambican doctors practice, and to extend the reach and increase the quality of health services [88,89].

Those professionals who ***are ***available often prefer to live and work in Maputo, as the school system quality and career opportunities for their families are greater. Incentives for doctors and nurses to live in rural areas have been implemented, but the number choosing to remain in remote areas is still small. Additional incentives must be provided, including quality education in rural areas for children, good housing for health care workers, and a more rewarding work environment. Task shifting of work from doctors to nurses, medical technicians, and community workers is also essential, as is a stemming of the tide of recruiting nurses and doctors from Africa to higher income Organization for Economic Co-operation and Development (OECD) countries [90-93].

### Treatment Challenges

Getting people to access ART is not straightforward, even when it is freely available. With high levels of stigma associated with HIV, openly taking life-long medication can present undesirable or even untenable social challenges, given the rarity with which chronic diseases are managed with continuous medication therapy, particularly in rural areas. The scaling up of HIV treatment in Mozambique has progressed at a slower rate than in neighboring Zambia, whose health challenges are similar to those in Mozambique [94,95]. Zambia is a more urbanized nation and never suffered from a colonial war of independence nor a civil war. Training, recruiting and retaining health care workers in remote/rural areas are part of Mozambique's chronic challenge. In 2004, Mozambique was believed to have 514 doctors, 3,954 nurses, and 2,229 midwives for a population at that time of ≈19 million [96]. By 2006, Mozambican health officials estimated that there were only 1.26 health care workers (of any profession) per 1,000 people [97]. The government has proposed a US$2 billion investment in long and short-term education programs to increase the number of doctors, medical technicians, nurses and others. Even with this investment, Mozambique will only have a projected 1.87 health care workers per 1,000 people by 2015 [98].

Barriers to prevention and treatment access are often limited by finances. Primarily donor funded, Mozambique's HIV treatment programs still face an uphill battle. A senior National Institutes of Health physician seconded to a high-level White House policy advisory position has stated that "...doubling or tripling PEPFAR's funding is not the best use of international health funding. In focusing so heavily on HIV/AIDS treatments, the United States misses huge opportunities" [99]. True to this no-more-money-for-ART philosophy, no substantial increase from base funding for HIV treatment from either the PEPFAR or the Global Fund has accompanied the WHO change in recommended treatment guidelines changing the routine start of ART from 250 to 350 CD4+ cells/μL [100]. Forty percent more persons should be on ART, but few in Mozambique will receive it. This challenge, in part due to the current financial crisis and donor fatigue, will limit the support provided for those HIV positive and for prevention interventions within the country.

## Conclusions

Mozambique has one of the highest HIV prevalence and incidence rates, showing few signs of abatement. Infrastructures are poor, due in part to colonial mismanagement of the country and due to the destruction of the few services that existed during the war of independence against the Portuguese and/or the civil war. Cultural norms around acceptability of multiple sexual partners and the low socio-economic status of women have not changed notably. Health care for people living with HIV/AIDS has become available nationwide, including for the rural population, but clinics offering services are often located far from villages and suffer from supply chain management issues. Stigma, social norms, and education must be addressed by directed interventions (increased educational opportunities, behavioral change campaigns, educational campaigns) aimed at overcoming and minimizing obstacles to health care in Mozambique. Building the health system infrastructure of Mozambique, including manpopwer, is essential for long term progress.

## Competing interests

The authors declare that they have no competing interests.

## Authors' contributions

All of the authors contributed to researching, constructing, and editing the final manuscript.

## Pre-publication history

The pre-publication history for this paper can be accessed here:

http://www.biomedcentral.com/1472-698X/10/15/prepub
